# Our Daughters

**Published:** 1878-07

**Authors:** 


### OUR DAUGHTERS.

As this country grows older, the necessity increases of each individual being able to earn a living. Hitherto, we could afford, in a measure, to allow our sons to grow up without the knowledge of any handicraft, as there were other avenues for employment; but already has it become important, in cities and large towns, that the daughters of a family should be able to earn something for the general sustenance of the household. Some give lessons in music, others teach school, most, too many, are driven to the heart-crush ing, health-destroying, and life-wasting stitch, stitch, stitch.

There seems to be a general repugnance against putting our daughters in public places, in shops, stores, and the like; and, as for making nurses, and chamber-maids, and waiters, and cooks of them, it is not to be thought of—yet awhile. But we must come to it at last. Other nations will cease to be able to supply us with hewers of wood and drawers of water—with carriage-drivers and menials for the household. The older nations fill these stations with their own poor; there is no sufficient reason why we should not do the same. That we should submit that our children should be nursed in their earlier years by those of a different religion, can only be accounted for in the existence of a false pride. The true wisdom of any denomination of Christians is, in giving the instruction and care of their children to those of a like faith with themselves.

In France, three-fifths of the females grown are under the necessity of doing something towards earning a livelihood.

It is very certain that the consciousness of not being able to make a support, casts many a girl on the street, compels others to marriages of policy, and takes from all, that independence of feeling, of character, and that self-reliance, which, of themselves, elevate, energize, and ennoble. Every year it is becoming less and less possible, even for the half of our daughters to marry men who can afford that they should do nothing towards earning a dollar. Hence, it is a true, a wise, and a high humanity to study out ways and means by which young girls can be placed in circumstances by which they can sustain themselves—something to fall back upon, in case of being thrown on their own resources, by orphanage, widowhood, or unfortunate marriages. A young widow of the city of New York, with a wise and humane charity, has inaugurated, by her influence and money, an establishment on Long Island for training young orphan girls in the art of horticulture, including the raising and preservation of vegetables, fruits, and flowers, the breeding of poultry, and everything connected with a farm life, which woman can do easily and well. If it is true that the man who rears a son without having him taught the means of earning a living, rears that child to large chances for the penitentiary and the gallows, it is not the less true, and is becoming daily more so, that the daughter who is ushered into womanhood without the knowledge and ability to earn a dollar by honorable means, is raised to the chances of an early death, or degradations worse than death itself.

